# Editorial: Dynamic cognition: empirical and theoretical insights from nonhuman primate studies in naturalistic conditions

**DOI:** 10.3389/fpsyg.2024.1432939

**Published:** 2024-06-26

**Authors:** Cheng Xue, Chih-Yang Chen, Xiaoguang Tian, Afonso C. Silva

**Affiliations:** ^1^Department of Neurobiology, University of Chicago, Chicago, IL, United States; ^2^Institute for the Advanced Study of Human Biology, Kyoto University, Kyoto, Japan; ^3^Department of Neurobiology, University of Pittsburgh, Pittsburgh, PA, United States

**Keywords:** nonhuman primate, higher cognitive function, natural behavior, automated behavioral testing, cognitive flexibility, animal models for disease

## Introduction

Nonhuman primates (NHPs), with their advanced cognitive capabilities, complex social interactions, and genomic closeness to humans, stand as pivotal models in neuroscience and biomedical research (Roelfsema and Treue, 2014). Through meticulously controlled experiments in constrained lab settings, NHP research over half a century has revealed critical neural mechanisms underlying cognitive functions and brain disorders (Gold and Shadlen, 2007; Scott and Bourne, 2022). Yet, we believe research with NHPs holds even greater potential as we begin to shift our research paradigm to truly take advantage of the animals' advanced, sophisticated behaviors and in naturalistic conditions. The present Research Topic features four articles on this front, exemplifying how the new NHP research paradigm led to important insights into complex animal cognition translatable to humans while simultaneously enhancing the welfare of the research animals involved.

## Emphasizing naturalistic conditions and cognitive phenomena

In contrast with the traditional approach, where constrained animals perform overlysimplified and abstract tasks, research in naturalistic conditions provides a more authentic reflection of NHPs' natural behaviors and social interactions, allowing more accurate characterization of the mechanism underlying cognitive processes. For instance, in one article of this Research Topic, Quintero et al. examined the vocal behaviors of sooty mangabeys in their natural foraging groups rather than in isolation. The results, which indicated that vocalizations increased in specific social contexts instead of direct food calls, suggest that complex social dynamics in the native environments of the animals is a significant factor in the evolution of their mode of communication (Quintero et al.). As social cognition related research like this gain increasing attention, it is becoming a prerequisite for studies to be carried out in naturalistic conditions, otherwise the findings would lose the most important relevance in behavior and therefore be far from conclusive.

## The promise of automated behavioral testing

However, transitioning from well-controlled rig-based experiments to such complex environments presents substantial methodological challenges, especially behavioral data analysis. In overcoming these challenges, the abundance of controlled data produced by advanced automated behavioral testing represents a significant breakthrough. Here, Cabrera-Moreno et al. discussed an automated, unsupervised training protocol that enabled long-tailed macaques to directly engage in a two-alternative choice task within their home enclosures. Similarly, Yurt et al. demonstrated a novel assessment of cognitive flexibility that challenged rhesus macaques to switch among multiple different tasks, demonstrating the capability to flexibly adapt to and from different rules. This approach produced highly consistent results with traditional rig-based experiments (Xue et al., 2022) but with much higher behavioral throughput at thousands of trials per session. The richness of the behavioral data is the key to the neuronal mechanisms underlying behavior. For instance, with enough behavioral data from the animal as a training set, it is possible to train artificial neural networks to reproduce the choice pattern of the animal, including the specific types of mistakes they make, thereby providing neurobiological hypotheses testable with more in-depth methods, such as electrophysiology (Xue et al., 2024). This integration of automated behavioral testing with AI, through methods like behavioral-data-driven RNNs, streamlines hypothesis testing—moving away from the problematic approach of sequentially testing intuition-based hypotheses one after another, until one shows statistical significance. This approach not only enhances the accuracy of AI models by using rich data sets from naturalistic settings but also automates hypothesis generation and reduces observer bias, thus ensuring more objective and reproducible outcomes.

## Establishing disease models with NHP

Furthermore, to overcome the cognitively implicated neuropsychiatric diseases, NHP models are essential for understanding mechanisms and developing potential remedies. Polyakova et al. provide an example of integrating pharmacological models with behavioral observations in naturalistic-like conditions, studying the impact of ketamine on eye movement characteristics of common marmosets. This approach simulates symptoms of schizophrenia, offering insights into how these primates process visual information under different conditions and providing a model for understanding psychiatric disorders (Polyakova et al.). With an NHP model of schizophrenia, a system or molecular level understanding of the disease can be expected to push forward the early diagnosis and intervention for tens of millions susceptible to it.

## Future directions and insights

As we refine NHP research in natural conditions, the potential for groundbreaking discoveries in NHP cognition and its applications to human cognitive studies continues to grow. In the future, the developments in new technology, such as automatic 3D behavioral tracking and analyses (Bala et al., 2020), wireless electrophysiological devices (Berger et al., 2020), and virtual reality (Castelvecchi, 2016), will likely expand the scope of what can be studied in NHPs. These technologies allow researchers to observe and analyze behavior in dynamic, unrestrained settings, offering a clearer window into the cognitive processes that naturally occur in health and disease.

Overall, by prioritizing naturalistic research settings and leveraging advanced automated testing technologies, we believe we are shifting toward a scientifically informing and ethically aware standard of research with NHPs. These approaches unlock profound new insights into primate cognition, demonstrating the indispensable value of NHPs in scientific innovation in the study of complex cognitive behaviors.

## Author contributions

CX: Writing – original draft, Writing – review & editing. C-YC: Writing – review & editing. XT: Writing – review & editing. AS: Writing – review & editing.
